# Lithological Mapping in High-Vegetation Areas Using Sentinel-2, Sentinel-1, and Digital Elevation Models

**DOI:** 10.3390/s25072136

**Published:** 2025-03-28

**Authors:** Yansi Chen, Genyuan Liu, Zhihong Song, Ming Li, Minhua Wang, Shuang Wang

**Affiliations:** 1 Center for Geophysical Survey, China Geological Survey, Langfang 065000, China; chenyansi@lzb.ac.cn (Y.C.); songzhihong2018@126.com (Z.S.); lming01@mail.cgs.gov.cn (M.L.); wangmh_geo@outlook.com (M.W.); xiaowang_ws@163.com (S.W.); 2 Technology Innovation Center for Earth Near Surface Detection, China Geological Survey, Langfang 065000, China

**Keywords:** lithology mapping, high-vegetation areas, RF, Sentinel-2, Senitnel-1, DEM

## Abstract

The extraction of lithological information in areas with high vegetation coverage presents numerous challenges, particularly in identifying concealed lithological features. This study focuses on a typical high-vegetation coverage area in Taiwan Province, China, utilizing multi-source data from Sentinel-2, Sentinel-1, and DEM, and using the Random Forest algorithm for lithological mapping. The results demonstrate that the optimal combination of Sentinel-2 and DEM significantly enhances the classification performance, achieving an overall accuracy (OA) of 84.30% and a Kappa coefficient of 0.83 in the validation set. Geological conditions have specific limiting effects on ecosystems, as spectral features (such as B2 and NDBI) and topographic features (such as elevation) contribute significantly to the classification results. This study provides valuable reference information for lithological information extraction in areas with high vegetation coverage.

## 1. Introduction

Lithology identification is crucial in geological research and resource exploration [1]. By recognizing different rock types and their distribution characteristics, we gain a deeper understanding of the Earth’s geological structure, the distribution of mineral resources, and surface evolution processes [2,3,4]. Moreover, lithological information is vital for mineral resource exploration, environmental geological assessments, and disaster prevention, making accurate lithology identification invaluable for geological investigations and related applications [5,6].

Traditional lithology identification relies primarily on manual investigations and visual interpretations, which can be time-consuming, labor-intensive, and heavily dependent on the experience of the investigators [7]. Due to constraints in manpower and time, traditional methods often struggle to achieve high precision in wide-area lithology identification [8]. These challenges are magnified in regions characterized by complex terrain and dense vegetation, where surface features are frequently obscured, and field access is often restricted [9]. Despite some advancements in the field, research specifically focused on lithology identification in areas with high vegetation coverage remains limited. The effective extraction of lithological information in such environments continues to present a substantial challenge, highlighting the critical need for continued methodological innovation and focused investigation.

Spectral imagery serves as the primary data source for lithology identification, as it reflects the spectral characteristics of surface materials [10]. However, atmospheric effects, the complexity of surface cover, and vegetation interference can limit the effectiveness of spectral images in lithology identification [11]. Consequently, radar imagery has garnered increased attention. Radar images can penetrate clouds and vegetation, providing critical information on surface roughness and dielectric constants, which enhances lithology identification [12,13]. Nonetheless, lithology identification is not solely dependent on spectral and radar images; it is also closely related to topographic relief. Therefore, Digital Elevation Models (DEMs) play an essential role in extracting lithological information [14]. Previous studies have shown that data fusion improves lithological classification, particularly in areas with dense vegetation. Combining optical and topographic data helps reduce vegetation interference, while integrating optical and radar data provides complementary spectral and structural information. These dual-source approaches have proven effective under complex surface conditions. However, most existing research has focused on dual-source data fusion—typically optical and DEM or optical and radar imagery—while comprehensive integration of all three data types (optical, radar, and topographic) remains relatively unexplored. The synergistic use of these data sources could offer a more holistic representation of lithological variability by capturing spectral, structural, and geomorphic characteristics simultaneously.

While various methods have been proposed for lithology identification, including visual interpretation and empirical judgment, these approaches heavily rely on manual experience and often result in low efficiency and limited accuracy [15]. More recent methods, such as machine learning techniques, offer the promise of improving recognition efficiency and accuracy by automatically extracting features from large datasets. Commonly used machine learning methods include Support Vector Machines (SVMs) [16], eXtreme Gradient Boosting (XGBoost) [17], and deep learning-based approaches such as convolutional neural networks (CNNs) [18,19]. However, these methods have significant weaknesses: SVMs are sensitive to kernel parameter selection and may perform suboptimally on large-scale datasets with high dimensionality [20], XGBoost requires computationally intensive hyperparameter tuning [21], and deep learning models, although promising, demand large training datasets and substantial computational resources, which are often unavailable in remote sensing applications with limited ground truth data [18].

In contrast, the Random Forest (RF) algorithm possesses several key advantages that make it particularly well-suited for lithological classification in scenarios characterized by limited field data and high input dimensionality. As an ensemble learning method, RF constructs multiple decision trees and combines their predictions via majority voting, thereby effectively reducing variance and reducing the risk of overfitting [22]. Additionally, RF is robust to high-dimensional data and parameter tuning, can handle missing values without preprocessing, and provides internal feature importance metrics, making it an ideal choice for lithology identification without the need for dimensionality reduction. Moreover, RF leverages Out-of-Bag (OOB) error estimation for internal validation, thus eliminating the necessity for a separate test set and enhancing both model efficiency and generalizability [23].

This study aims to evaluate the contribution of multi-source data to lithology mapping in areas with high vegetation coverage, employing RF as the primary classification algorithm. The main data used include Sentinel-2 spectral images, Sentinel-1 radar images, and Digital Elevation Models (DEMs). By integrating these multi-source data, we hope to improve the accuracy of lithology identification, particularly in regions with dense vegetation. The research seeks to provide an effective solution for lithology extraction in complex environments and offers valuable reference for related work in mineral resource exploration and geological disaster prevention.

## 2. Materials and Datasets

### 2.1. Study Area

**Figure 1:**
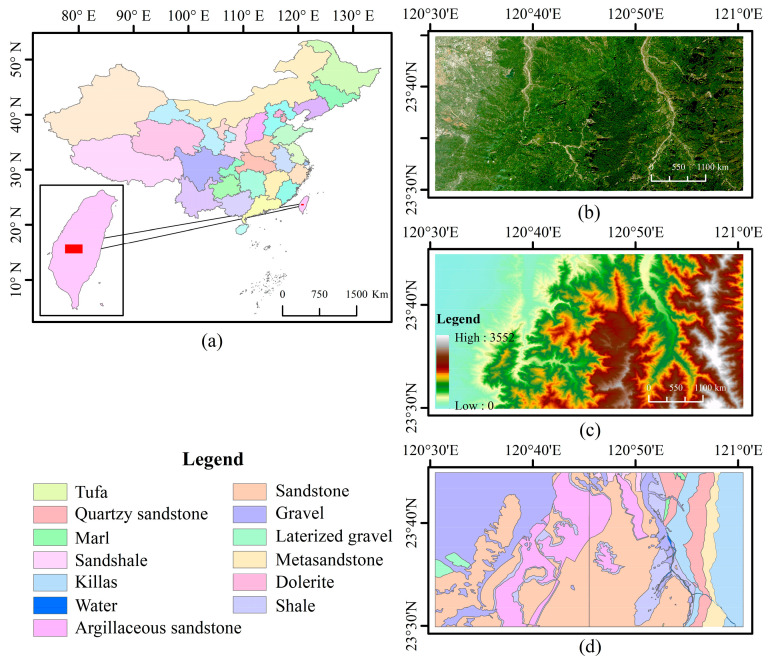
Overview of the study area: (**a**) administrative map, (**b**) Sentinel-2 imagery; (**c**) SRTM DEM from Google Earth Engine Platform; (**d**) geologically modified map created by professional geologists.

The study area is located in Taiwan Province, China, with its specific distribution administrative zoning map shown in Figure 1a. The area features a complex distribution of rock bodies, representing a strong geological diversity. The main rock types within the study area include ignimbrite, slate, diabase, and marl, reflecting a variety of lithological characteristics and geological structural backgrounds. The region is characterized by a wide range of rock types, each with distinct origins and depositional environments, providing a rich array of samples and data support for geological research and mineral resource exploration. However, the area also exhibits high vegetation coverage (Figure 1b) and a complex topography (Figure 1c), which poses certain challenges for lithological classification.

### 2.2. Remote Sensing Image and Preprocessing

#### 2.2.1. Sentinel-2 Imagery and Preprocessing

Sentinel-2 (S2) is a pair of multispectral satellites launched by the European Space Agency (ESA), consisting of Sentinel-2A and Sentinel-2B, which were launched in June 2015 and March 2017, respectively [24]. The imagery provides a revisit cycle of five days, covering 13 spectral bands ranging from visible light to shortwave infrared (wavelength range: 443 nm to 2190 nm). In this study, bands B2, B3, B4, B5, B6, B7, B8, B8A, B11, and B12 were used. These bands provide spatial resolutions of 10 m (visible and near-infrared), 20 m (red-edge and shortwave infrared), and 60 m (atmospheric correction). The S2 L2A (Level-2A) dataset utilized in this study is derived from the official preprocessing steps conducted by the data provider. These steps include atmospheric correction, cloud and shadow masking, terrain adjustment, illumination and viewing geometry normalization, as well as radiometric calibration and quantization. Through these processes, the data are transformed into surface reflectance values, enabling direct quantitative analysis. Thus, employing Sentinel-2 L2A imagery from 2023 ensures high-quality data while substantially reducing the complexity of preprocessing workflows, thereby enhancing its suitability for classification and feature identification tasks.

To enhance Sentinel-2 imagery quality and reduce cloud interference, a median composite approach was applied [23]. First, pixels with a cloud probability below 60% were filtered using the cloud probability layer. The remaining cloud-free pixels were then used to generate a standardized spectral composite. Finally, a median calculation was performed across the selected images to mitigate temporal variation effects, producing a single, stable, and cloud-free image. For further feature extraction, several common indices were calculated, including NDBI (Normalized Difference Building Index), NDWI (Normalized Difference Water Index), LSWI (Land Surface Water Index), EVI (Enhanced Vegetation Index), and GCVI (Green Chlorophyll Vegetation Index) [23].

#### 2.2.2. Sentinel-1 Imagery and Preprocessing

Sentinel-1 (S1), launched by ESA in 2014, consists of radar satellites (S1A and S1B) for acquiring all-weather, global synthetic aperture radar (SAR) imagery, enabling surface observation under cloud and vegetation cover [25]. S1 uses C-band radar with a 6-day revisit cycle. This study employs the Interferometric Wide (IW) mode, providing 10 m spatial resolution. The median composite of S1 imagery from 2023 was computed, similar to the approach employed for Sentinel-2 data, in order to minimize anomalies caused by temporal variations and noise.

The refined Lee filter was applied to preserve edges and details while reducing noise based on neighborhood statistics as S1 imagery is often affected by speckle noise [26]. The VV and VH polarization bands were extracted, and the VV/VH ratio index was calculated, serving as a key indicator of surface roughness and other features.

#### 2.2.3. SRTM DEM

The Digital Elevation Models (DEMs) serve as a crucial data source for topographic information, offering surface relief details. This study uses the 30 m resolution Shuttle Radar Topography Mission (SRTM) DEM [27]. To effectively mitigate local high-frequency noise, Gaussian smoothing techniques were applied to refine the DEM dataset [28]. Subsequently, utilizing this refined DEM, elevation, slope, aspect, and topographic indices (*SR*, *HI*, and *SI*) were computed to facilitate a comprehensive extraction of surface features and lithology analysis. Here, slope is expressed in degrees, indicating terrain steepness, while the Surface Index (*SI*) increases with surface irregularity—higher SI values reflect more pronounced deformation.

(1) SR=1/cos(slop)

(2) $$HI=(H_{mean}-H_{min})/(H_{max}-H_{min})$$

(3) $$SI=\left(\frac{HI-{HI}_{min}}{{HI}_{max}}\right)\times \left(\frac{H-H_{min}}{H_{max}}\right)-(\frac{SR-(1+{SR}_{min})}{{SR}_{max}})$$

where $H_{mean}$, $H_{min}$, and $H_{max}$ represent the mean, minimum, and maximum elevations, respectively, calculated within a 3 × 3 moving window.

Finally, bilinear interpolation was used to unify the sampling of all the above data to a spatial resolution of 10 m, ensuring that different data sources have a consistent spatial scale. After completing the resampling, fusion processing was performed on S2, S1, and DEM, resulting in four combined datasets: S2+S1, S2+DEM, S1+DEM, and S2+S1+DEM.

### 2.3. Sample Dataset

The sample data are derived from pre-existing geological maps. Based on the geological conditions of the study area, experts refined the geological map and presented the revised lithological classification map in Figure 1d. A comprehensive categorization of 11 lithological types was established, encompassing Tuff, Quartz sandstone, Marl, Sandshale, Slate, Sandstone, Gravel, Laterized gravel, Metasandstone, Dolerite, and Argillaceous sandstone. In addition to these classifications, a new category named ‘Water Body’ was introduced with a random collection of 200 sample points for each type.

## 3. Method

**Figure 2:**
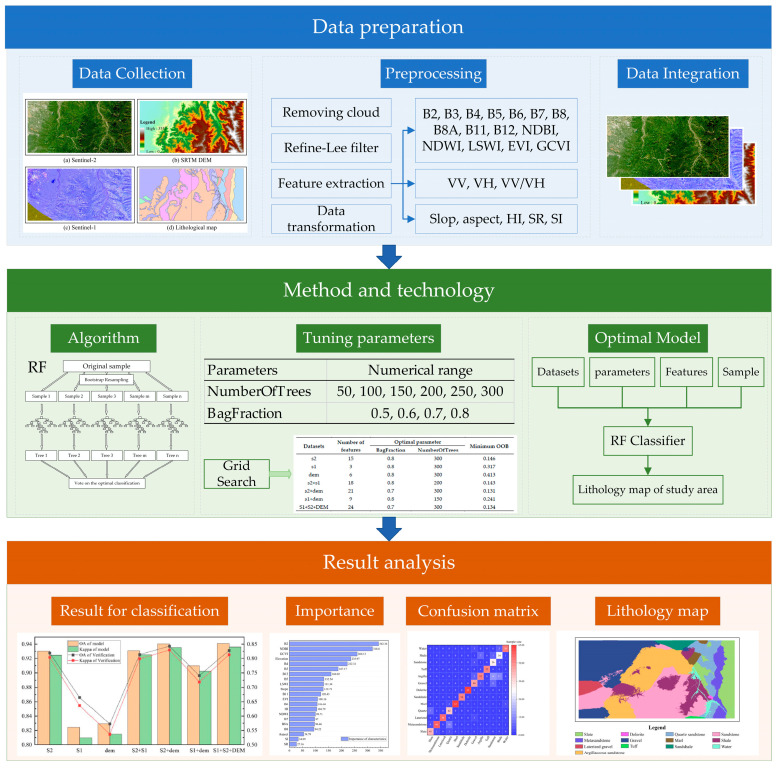
Technical flow chart.

The overall technical workflow (Figure 2) entails the utilization of the Random Forest algorithm for lithological mapping in the study area, employing six different data combinations (S2, S1, DEM, S2+S1, S2+DEM, and S1+S2+DEM). To fully leverage the information content of each data source, the classification scheme directly utilized all available feature variables without dimensionality reduction. Specifically, Sentinel-2 contributed 15 variables, including 10 spectral bands and 5 derived indices, Sentinel-1 provided three radar backscatter-related features (VV, VH, and VV/VH), and the DEM dataset offered six topographic variables (elevation, slope, aspect, SR, HI, and SI). During the model tuning phase, hyperparameter optimization was conducted using Out-of-Bag (OOB) error to evaluate various combinations of model configurations. The key hyperparameters adjusted included the number of trees, the number of features considered at each split, and the fraction of input data sampled per tree. The optimization process aimed to minimize the OOB error while ensuring the model’s ability to generalize effectively on unseen data. After determining the optimal settings, the model was trained using optimized parameters, and accuracy was validated using a set of validation samples. Ultimately, precise lithological classification was achieved in areas characterized by high vegetation coverage based on the optimal fusion of multi-source data. This comprehensive process effectively showcases the advantages of multi-source data fusion in enhancing geological classification accuracy.

Random Forest is an ensemble learning technique that achieves classification or regression by constructing multiple decision trees and aggregating their predictions through voting or averaging [29]. The advantages of this method include high accuracy, strong resistance to overfitting, and the ability to handle missing values and high-dimensional features [30]. Additionally, Random Forest can assess feature importance, helping us to identify the variables that have the greatest impact on prediction outcomes [31].

One significant advantage of the algorithm is the Out-of-Bag (OOB) error, which uses samples not trained by a particular tree to make predictions, providing an unbiased estimate of model performance [32]. This approach eliminates the need for cross-validation, making the model training process more efficient. The OOB can be monitored in real time during model training, aiding in the optimization of model parameters and enhancing its generalization ability.

## 4. Results and Discussion

### 4.1. Parameter Adjustment

To ensure optimal model performance, we employed a grid search strategy to fine-tune key hyperparameters for the RF classifier, specifically the number of decision trees (NumberOfTrees) and the sampling proportion of the training data (BagFraction). The grid search was performed by systematically evaluating combinations of NumberOfTrees ranging from [50, 100, 150, 200, 250, 300] and BagFraction values set at [0.5, 0.6, 0.7, 0.8], with the OOB serving as the evaluation criterion. For each dataset scenario, the parameter combination that resulted in the lowest OOB error was identified as the optimal configuration. Furthermore, the number of variables randomly selected at each split was set to the square root of the total number of input features, following standard practice in RF implementations. Through the systematic optimization of these parameters, the RF model exhibited significantly enhanced generalization capability and effectively minimized classification error. As illustrated in Table 1, optimal combinations of input features, coupled with meticulously tuned hyperparameters, played a crucial role in achieving superior lithological classification accuracy.

When considering individual features, such as S2, S1, and DEM, separately, it was observed that S2 exhibited the lowest OOB value (0.1459), while S1 and DEM displayed higher OOB values (0.3171 and 0.4133, respectively). However, when combining multiple features together, it was found that the combination of S2+DEM achieved the lowest OOB value (0.1315) under optimized parameter settings (bagFraction = 0.7; numberOfTrees = 300), indicating its superior capability in capturing patterns within the data. Furthermore, both S1+DEM and S1+S2+DEM combinations demonstrated relatively good performance with respective OOB values of 0.2409 and 0.1337.

Generally, increasing the number of features and utilizing dataset combinations tend to reduce errors, as reflected in the decrease in OOB values with more features. Additionally, combining multiple datasets consistently leads to lower OOB errors. Moreover, fine-tuning parameters like BagFraction and NumberOfTrees can further enhance model performance.

### 4.2. Classification Results of Different Data Combinations

**Figure 3:**
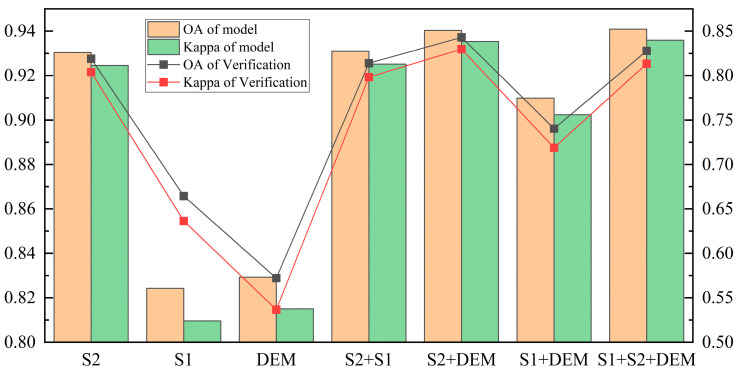
Comparison of classification accuracy based on different data combinations.

The classification results obtained from different data sources were evaluated. Based on the results (as depicted in Figure 3), when solely utilizing S2 features, the model achieved an overall accuracy (OA) of 93.04%, with a validation OA of 81.90% and a validation Kappa coefficient of 0.80, indicating commendable performance. Conversely, the classification outcomes for S1 and DEM exhibited relatively subpar performance, particularly for DEM alone, which demonstrated a validation OA of 57.22% and a Kappa coefficient of 0.54, suggesting limited predictive capability when relying solely on DEM data.

The optimal combination of spectral information and terrain features in the data combination S2+DEM enhances the comprehensiveness of input for the model, thereby improving classification accuracy and robustness. The model achieved an OA of 94.03% and a verification OA of 84.30%, with a verification Kappa value of 0.83, indicating significant improvements in classification accuracy. Furthermore, the combination of S1+S2+DEM also demonstrated excellent performance, yielding a model OA of 94.09%, verified OA of 82.78%, and verified Kappa value of 0.81, further confirming the advantages offered by multi-source data integration. Compared with previous studies, such as Othman et al. (2017) [33], which achieved an OA of approximately 80% using integrated spectral, spatial, and morphometric features with Random Forest, and even lower accuracy (~74%) using SVM, the proposed method shows notably improved performance.

**Figure 4:**
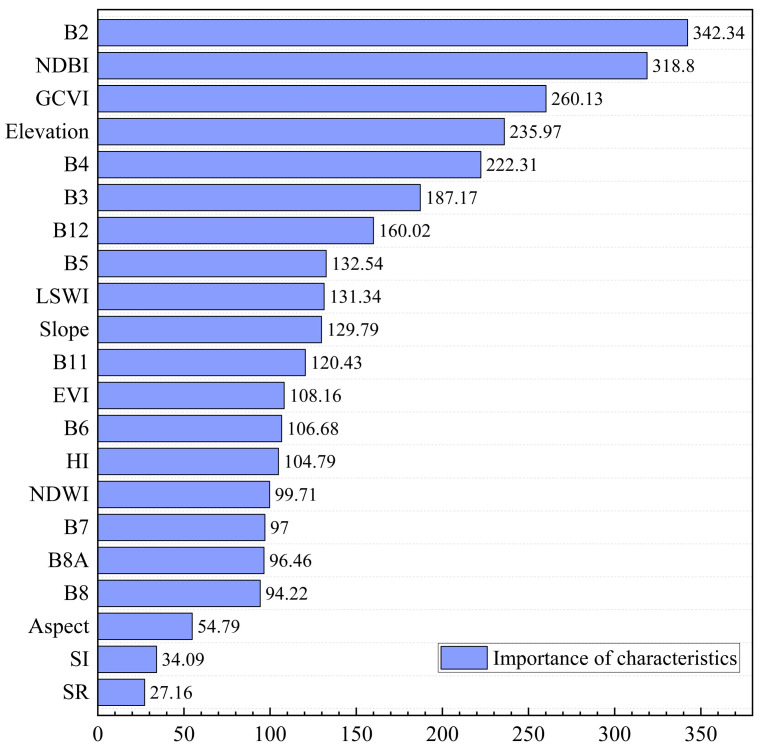
Importance evaluation of characteristic variables from S2+DEM combined data.

Comprehensive analysis shows that the combination of S2 and DEM performs best across all indicators, validating the importance of multi-source data fusion in improving classification accuracy. The importance of various feature variables in this combined dataset is shown in Figure 4, where spectral features and topographic features have varying degrees of influence on model classification. Inspired by SHAP-based interpretability frameworks, we further consider not only the ranking but also the directional contribution of features [17]. Specifically, B2 (342.34) provides a strong positive contribution to distinguishing lithologies with distinct reflectance in the blue band, while NDBI (318.80) and GCVI (260.13) support the classification of urbanized or vegetated surfaces, respectively. Elevation (235.97), as the most influential topographic feature, often contributes to separating rock types distributed at different altitudes. Additionally, B4, B3, and B12 also contribute positively by capturing vegetation vigor and shortwave-infrared absorption characteristics, which are critical in differentiating lithological units under dense vegetation. Conversely, S1 (34.09) and SR (27.16) exhibit low significance, indicating their relatively limited contribution to the classification.

**Figure 5:**
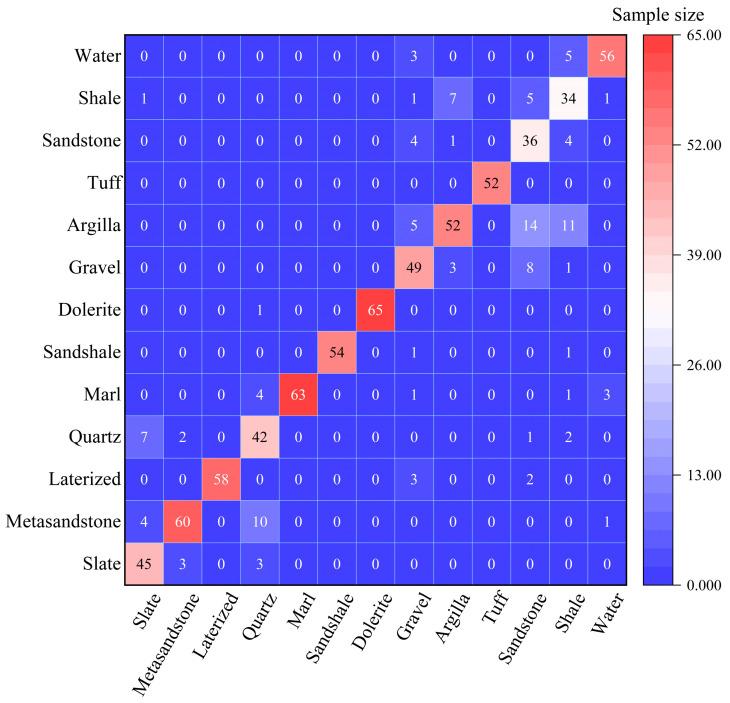
The confusion matrix derived from the model validation process. The x-axis represents the category predicted by the model, the y-axis represents the category of the actual observation, and the diagonal represents the correct interpretation. Here, “Quartz” refers to “Quartz sandstone”, and “Argilla” refers to “Argillaceous sandstone”.

Furthermore, the confusion matrix obtained statistically for the validation sample is presented in Figure 5. The analysis of the confusion matrix reveals a high level of classification accuracy across most categories. Notably, Slate, Marl, and Metasandstone samples were almost entirely classified correctly, while Water, Gravel, and Laterized gravel were accurately predicted as well. However, there is a significant degree of confusion among certain lithologic classes, particularly those with similar composition or appearance, such as Slate and Quartz Sandstone, or Quartz Sandstone and Metasandstone. This confusion likely stems from similarities in rock composition and particle structure. While the classification of water generally demonstrates accuracy, occasional misclassifications do occur, such as instances where Metasandstone and Marl are incorrectly predicted as water. These misclassifications may be influenced by the surrounding geological environment or classification boundaries.

### 4.3. Lithological Mapping

**Figure 6:**
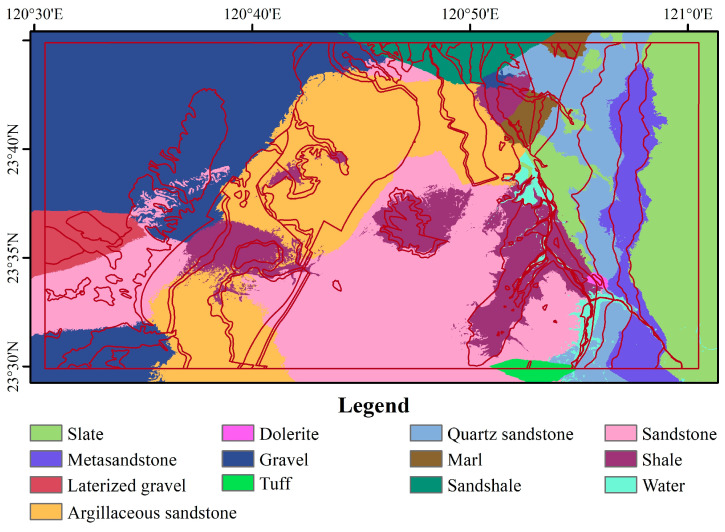
Lithological mapping based on the S2+DEM classification results, overlaid with the existing geological map outlines.

Using the optimal data combination (S2 and DEM) and the Random Forest algorithm, effective identification and classification of various lithologies were achieved, as shown in Figure 6. A comparison between the classification results and the existing geological map reveals that the outcomes are satisfactory, with the spatial distribution trends of lithologies closely matching actual conditions. The response of lithology types to topography is notable—Metasandstone is distributed in steeper areas, consistent with findings from other scholars [2]. Sandstone is found at higher elevations in the Three Gorges Group (central-southern part of the study area) with high vegetation coverage, indicating that lithology, soil characteristics, moisture conditions, microclimate, and plant adaptability collectively create a suitable environment for vegetation growth.

Additionally, the sedimentary environment of the Argillaceous sandstone area gradually becomes gentler, allowing finer sediment to settle in mid-elevation regions. Particularly in the northwest of the study area, Gravel is located in alluvial layers where the terrain is relatively flat (Figure 1c) and vegetation coverage is low (Figure 1b), indicating that sedimentary environment and soil conditions impose significant limitations on vegetation growth in this area. This further confirms the restrictive impact of lithological characteristics on the ecological environment [9,34].

Comparing these classification results with existing geological maps reveals several differences, primarily due to spatial resolution, data sources, and surface cover. The 10 m resolution and spectral characteristics of Sentinel-2 limit the capture of fine details, leading to discrepancies between the classification results and traditional geological maps, especially in areas with complex geology or dense vegetation. In regions with dense vegetation, remote sensing imagery may struggle to penetrate the vegetation layer, potentially causing the misclassification of lithology types. Additionally, in geologically complex areas, particularly along fault zones or lithological transitions, the RF classification may miss subtle lithological variations, while geological maps, based on field surveys and actual geological conditions, provide more detailed descriptions of these phenomena.

However, the remote sensing classification results not only complement existing geological maps but also offer new insights into lithological distribution, highlighting the potential of remote sensing in geological research. The integration of DEM-based terrain information further reveals the impact of topography on lithological distribution, providing a more refined classification.

## 5. Conclusions

This study employed the optimal data combination (S2 and DEM) and utilized the Random Forest algorithm to successfully achieve high-precision lithological classification in areas with dense vegetation. The experimental results indicate that the optimal combination significantly enhanced the model’s classification performance, with the overall accuracy (OA) of the validation set reaching 84.30% and a Kappa coefficient of 0.83, demonstrating the effectiveness of this method in lithological identification.

Feature importance analysis revealed that spectral features (e.g., B2 and NDBI) as well as topographic features (e.g., elevation) made substantial contributions to the model’s classification outcomes, underscoring the crucial role of multi-source data fusion in geological exploration. By integrating diverse features, our model comprehensively captures lithological distribution characteristics while enhancing classification robustness.

This study addresses lithological classification in vegetated areas using spectral and topographic data, providing a methodological basis for related geological applications. Nonetheless, some limitations should be acknowledged. The model primarily incorporates spectral and terrain variables, without considering additional environmental factors such as hydrology, land surface temperature, or geological structure, which may affect lithological differentiation. In addition, while the Random Forest algorithm performs well with multi-source and high-dimensional data, its ability to represent complex and nonlinear spatial patterns is limited compared to more advanced deep learning methods. Subsequent research will investigate the influence of different coverage types (such as forests, grasslands, and wetlands) and different coverage degrees (ranging from sparse to dense) on lithology classification outcomes. Further integration of hydrological, climatic, and structural geological information will be considered to expand the feature space. The application of more sophisticated algorithms, such as convolutional neural networks (CNNs) or transformer-based models, will also be explored to enhance classification performance in geologically complex regions. These developments are expected to support the construction of more robust and generalizable lithological mapping approaches.

## Figures and Tables

**Table 1:** Parameter adjustment results of different combinations of data.

Datasets	Number of Features	Optimal Parameter		Minimum OOB
		BagFraction	NumberOfTrees	
S2	15	0.8	300	0.146
S1	3	0.8	300	0.317
DEM	6	0.8	300	0.413
S2+S1	18	0.8	200	0.143
S2+DEM	21	0.7	300	0.131
S1+DEM	9	0.8	150	0.241
S1+S2+DEM	24	0.7	300	0.134

## Data Availability

The dataset is available upon request from the authors.
